# Supplementary material for: HIVprotI: an integrated web based platform for prediction and design of HIV proteins inhibitors
Source: J Cheminform. 2018 Mar 9;10:12. doi: 10.1186/s13321-018-0266-y (PMC5845081; doi:10.1186/s13321-018-0266-y)
Supplement: Supplementary file 2 — Additional file 2. Source code of HIVProtI web server. [file 13321_2018_266_MOESM2_ESM.zip › HIVprotI_Source-code/hivprotI/weka-3-6-6/doc/weka/experiment/Compute.html]

Compute


---


|  |  |  |
| --- | --- | --- |
| **Overview** \| **Package** \| **Class** \| **Tree** \| **Deprecated** \| **Index** \| **Help** \| **Weka's home** | |  |
| **PREV CLASS**   **NEXT CLASS** | **FRAMES**    **NO FRAMES**     **All Classes** |  |
| SUMMARY: NESTED \| FIELD \| CONSTR \| METHOD | DETAIL: FIELD \| CONSTR \| METHOD |  |


---


## weka.experiment Interface Compute

**All Superinterfaces:**: java.rmi.Remote

**All Known Implementing Classes:**: RemoteEngine

---

``` public interface Compute extends java.rmi.Remote ```

Interface to something that can accept remote connections and execute
a task.

**Version:**
:   $Revision: 1.5 $

**Author:**
:   Mark Hall (mhall@cs.waikato.ac.nz)

---

| **Method Summary** | |
| --- | --- |
| `java.lang.Object` | `checkStatus(java.lang.Object taskId)`             Check on the status of a `Task` |
| `java.lang.Object` | `executeTask(Task t)`             Execute a task |

**Method Detail**

### executeTask

```
java.lang.Object executeTask(Task t)
                             throws java.rmi.RemoteException
```

:   Execute a task

    :   **Parameters:**: `t` - Task to be executed **Returns:**: a unique ID for the task **Throws:**: `java.rmi.RemoteException` - if something goes wrong.

---


### checkStatus

```
java.lang.Object checkStatus(java.lang.Object taskId)
                             throws java.lang.Exception
```

:   Check on the status of a `Task`

    :   **Parameters:**: `taskId` - the ID for the Task to be checked **Returns:**: the status of the Task **Throws:**: `java.lang.Exception` - if an error occurs


---


|  |  |  |
| --- | --- | --- |
| **Overview** \| **Package** \| **Class** \| **Tree** \| **Deprecated** \| **Index** \| **Help** \| **Weka's home** | |  |
| **PREV CLASS**   **NEXT CLASS** | **FRAMES**    **NO FRAMES**     **All Classes** |  |
| SUMMARY: NESTED \| FIELD \| CONSTR \| METHOD | DETAIL: FIELD \| CONSTR \| METHOD |  |


---
